# Protective effects of hydrogen enriched saline on liver ischemia reperfusion injury by reducing oxidative stress and HMGB1 release

**DOI:** 10.1186/1471-230X-14-12

**Published:** 2014-01-12

**Authors:** Yantao Liu, Liqun Yang, Kunming Tao, Marcela P Vizcaychipi, Dafydd M Lloyd, Xuejun Sun, Michael G Irwin, Daqing Ma, Weifeng Yu

**Affiliations:** 1 Department of Anesthesia & Intensive Care, Eastern Hepatobiliary Surgical hospital, the Second Military Medical University, Shanghai 200433, China; 2 Anaesthetics, Pain Medicine & Intensive Care, Department of Surgery and Cancer, Imperial College London, Chelsea and Westminster Hospital, London SW10 9NH, UK; 3 Department of Diving Medicine, Faculty of Naval Medicine, the Second Military Medical University, Shanghai 200433, China; 4 Department of Anaesthesiology, University of Hong Kong, Queen Mary Hospital, Hong Kong K424, China

**Keywords:** Hepatology, Ischemia/reperfusion injury, Inflammatory mediators, Oxidant stress

## Abstract

**Background:**

The nuclear protein high-mobility group box 1 (HMGB1) is a key trigger for the inflammatory reaction during liver ischemia reperfusion injury (IRI). Hydrogen treatment was recently associated with down-regulation of the expression of HMGB1 and pro-inflammatory cytokines during sepsis and myocardial IRI, but it is not known whether hydrogen has an effect on HMGB1 in liver IRI.

**Methods:**

A rat model of 60 minutes 70% partial liver ischemia reperfusion injury was used. Hydrogen enriched saline (2.5, 5 or 10 ml/kg) was injected intraperitoneally 10 minutes before hepatic reperfusion. Liver injury was assessed by serum alanine aminotransferase (ALT) enzyme levels and histological changes. We also measured malondialdehyde (MDA), hydroxynonenal (HNE) and 8-hydroxy-guanosine (8-OH-G) levels as markers of the peroxidation injury induced by reactive oxygen species (ROS). In addition, pro-inflammatory cytokines including TNF-α and IL-6, and high mobility group box B1 protein (HMGB1) were measured as markers of post ischemia-reperfusion inflammation.

**Results:**

Hydrogen enriched saline treatment significantly attenuated the severity of liver injury induced by ischemia-reperfusion. The treatment group showed reduced serum ALT activity and markers of lipid peroxidation and post ischemia reperfusion histological changes were reduced. Hydrogen enriched saline treatment inhibited HMGB1 expression and release, reflecting a reduced local and systemic inflammatory response to hepatic ischemia reperfusion.

**Conclusion:**

These results suggest that, in our model, hydrogen enriched saline treatment is protective against liver ischemia-reperfusion injury. This effect may be mediated by both the anti-oxidative and anti-inflammatory effects of the solution.

## Background

The pathophysiology of hepatic ischemia reperfusion injury (IRI) is multifactorial and involves direct cellular damage, microcirculatory failure and an inflammatory response to tissue damage that culminates in organ dysfunction and failure [1,2]. However, studies have shown that the oxidative stress of IRI has a causal role. Cytotoxic free radicals attack lipids, proteins and nucleic acids within the cell, resulting in impaired mitochondrial function and increased lipid peroxidation. Substantial evidence exists to suggest that the initial production of reactive oxygen species (ROS) and endogenous Damage Associated Molecular Pattern Molecules (DAMPs) have also been implicated. The subsequent activation of several molecular and signaling cascades leads to cellular damage and an imbalance between pro and anti-inflammatory responses [3,4].

High Mobility Group Box 1 (HMGB1) is an endogenous damage associated molecule known to participate in nucleosome stabilization and regulation of transduction [5]. Originally implicated as a later mediator of sepsis [6], recent work [7,8] has shown that HMGB1 is an early mediator of injury and inflammation in liver IRI, and demonstrates a rapid increase in plasma levels following tissue reperfusion. Inhibition of HMGB1 release or application of anti-HMGB1 or HMGB1-receptor antagonist have been shown to reduce cytokine expression and preserve hepatic function in animal models, indicating that manipulation of HMGB1 may be a therapeutic target in hepatic IRI [7]. HMGB1 release following liver ischemia is triggered by the production of reactive oxygen species that activate a Toll-like receptor 4-dependent pathway mediated by calcium signaling [8]. Thus, anti-oxidant strategies for inhibiting HMGB1 release may be of therapeutic value in the prevention and treatment of hepatic IRI.

Administration of hydrogen gas has been reported to attenuate IRI in multiple organs [9-13], and to selectively reduce cytotoxic Oxygen radicals, sparing other free radicals with vital physiological roles [14]. Recently, for convenient and safe clinical hydrogen administration, hydrogen-enriched saline has been developed and the protective effects of Hydrogen-rich Saline against neuronal, intestinal and myocardial [15-18] ischemia-reperfusion injuries have already been well documented. It remains unclear, however, if hydrogen has an effect on HMGB1 in liver IRI. The present study was performed to investigate the possible therapeutic effects of hydrogen-enriched saline solution on liver IRI, and test the hypothesis that this solution confers protection against IRI by reducing oxidative stress and inhibiting the inflammatory response through modulation of HMGB1 production.

## Methods

### Experimental groups

**Figure 1:**
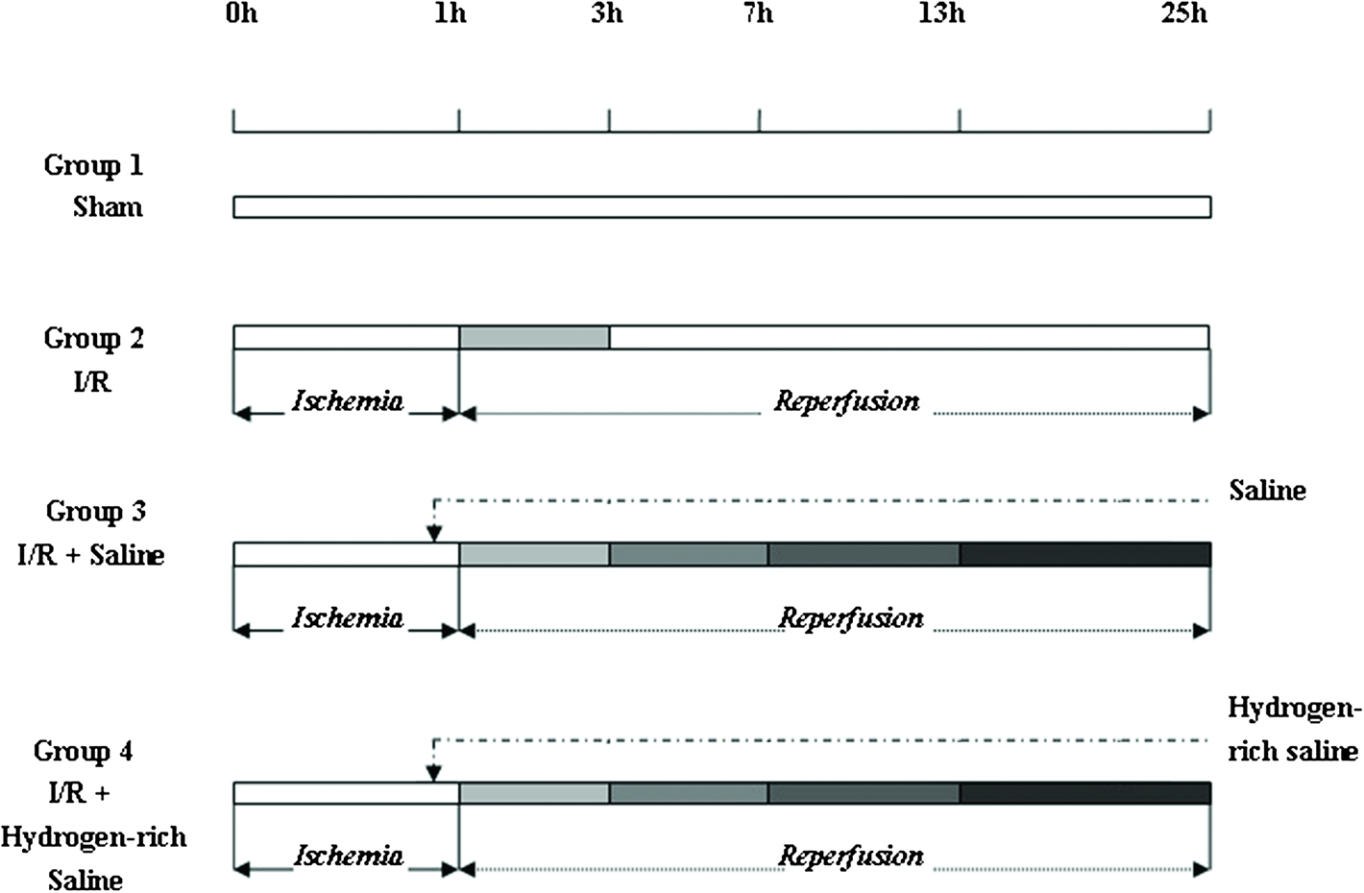
**Schematic illustration of the experimental protocol.** Sham: laparotomy and dissection of the portal vein but not clamping; I/R group: Ischemia was induced in the median and left lateral hepatic lobes for 1 hr, followed by 2 hour period of reperfusion; Normal saline + I/R group: Saline 10ml/kg injected intraperitoneally 10 minutes before hepatic reperfusion, followed by 2, 6, 12, 24 hour period of reperfusion; Hydrogen-enriched saline + I/R group: 1% hydrogen enriched saline (2.5, 5 or 10ml/kg) injected intraperitoneally 10 minutes before hepatic reperfusion, followed by 2, 6, 12, 24 hour period of reperfusion.

The study was performed in accordance with our institutional guidelines on the use of live animals for research and the experimental protocol was approved by the Animal Care and Use Committee of the Second Military Medical University, Shanghai, China. Male Sprague-Dawley rats weighing 250–300 g were housed in groups of 3–4 per cage in a temperature controlled environment of 22°C and an alternating 12-h light/12-h dark cycle. Animals were allowed free access to food and water until the night before anesthesia. 144 rats were randomly divided into four groups, as illustrated in Figure 1: Sham (n = 8), laparotomy and dissection of the portal vein but not clamping; Ischemia-reperfusion (I/R group, n = 8); Normal saline + I/R group (control group, n = 32) and hydrogen-enriched saline + I/R group (therapeutic group, n = 96, 32 per subgroup). Saline (10 ml/kg) or hydrogen enriched saline (2.5, 5 or 10 ml/kg) was injected intraperitoneally 10 minutes before hepatic reperfusion.

### Hydrogen enriched gas saline preparation

Supersaturated hydrogen gas saline solution was prepared by a method and with apparatus that has been described previously [19]. Briefly, hydrogen was dissolved in 0.9% saline for 2 h under high pressure (0.4 MPa) to the supersaturated level using hydrogen-rich water producing apparatus of our own design. The saturated hydrogen saline was stored under atmospheric pressure at 4°C in an aluminum bag without dead space. Hydrogen-rich saline was freshly prepared every week to ensure a constant Hydrogen concentration exceeding 0.6 mM.

### The model of liver ischemia-reperfusion

A model of segmental (70%) hepatic ischemia was used in the current study as previously described [20]. Briefly, a midline laparotomy was performed under surgical anesthesia using intra peritoneal pentobarbital (50 mg/kg) and 10% chloral hydrate (3 ml/kg). The portal triad (hepatic artery, portal vein, and bile duct) to the left anterior and median hepatic lobes were carefully occluded using vascular clamps to produce partial (70%) hepatic ischaemia. After 60 minutes the clamps were removed to allow organ reperfusion. The abdominal wound was then closed with sutures. Rectal temperature was maintained between 36-37°C by a heating lamp throughout all procedures.

### Sample collection

The animals were given a further dose of pentobarbitone (50 mg) before being sacrificed by exsanguination. The harvest time points were at 2 hrs post reperfusion in the sham and I/R group and 2, 6, 12, or 24 hours post reperfusion in the control and each therapeutic group respectively (n = 8 at each time point). The vena cava was opened and 3-5 ml blood collected in sterile syringes without anticoagulant and centrifuged to separate the serum. The serum samples were stored at -20°C for later batch analysis of hepatic function and cytokine assay.

The liver was perfused with cold saline through the portal vein. Ischemic left hepatic tissue samples were collected and specimens were either: 1) fixed in 10% formalin and embedded in paraffin for histological studies; or 2) immediately frozen in isopentane and liquid nitrogen, then stored at -80°C for later analysis.

### Serum alanine aminotransferase (ALT) measurement

ALT levels in serum were determined using a commercially available biochemical analyser (Model 7600, Hitachi Co, Tokyo, Japan) and expressed in IU/l.

### Hepatic malondialdehyde (MDA) measurement

Hepatic MDA levels were determined using an MDA-532 Assay kit (Jiancheng, Nan Jing, China). Briefly, frozen liver tissue was homogenized and boiled in a solution containing glacial acetic acid, thiobarbituric acid and NaCl buffer. After cooling to room temperature, the mixture was centrifuged at 1500 rpm/min for 15 min. MDA reacts with thiobarbituric acid forming a solution pink in colour. MDA was quantified in the supernatant by spectrophotometry (UV752, Shanghai, China) at a wavelength of 532 nm using a method described by Ohkawa et al. [21].

### Real-time RT-PCR

Total RNA was extracted from the liver using the Trizol reagent (Takara Bio Inc, Tokyo, Japan) as described in the manufacturer's instructions. TNF-α and IL-6 mRNA were quantified in duplicate by SYBR green two-step, real-time RT-PCR, as described by Tsung et al. [7]. Briefly, following removal of potentially contaminant DNA using DNase I (Invitrogen), 1 μg of RNA from each sample was used for reverse transcription with oligo dT (Invitrogen) and Superscript II (Invitrogen) to generate first-strand cDNA. The PCR reaction mixture was prepared using SYBR green PCR Master Mix (Applied Biosystems) using primers as follows: TNF-α 5′-CCCGGAATGTCGATGCCTGAGTG-3′, and 5′-CGCCCCGGCCTTCCAAATAAAT-3′; IL-6 5′-TCTCGAGCCCACCAGGAACG A-3′ and 5′-AGGGAAGGCAGTGGCTGTCA-3′. Thermal cycling conditions were 10 min at 95°C, followed by 40 cycles of 95°C for 15 s and 60°C for 1 min on a sequence detection system (ABI PRISM 7000; Applied Biosystems). Each expression gene was normalized with GAPDH mRNA using a Delta-Delta CT method.

### Western blotting

Frozen liver tissues were suspended in ice-cold cell lysis buffer (Beyotime Chemical Co, China.) that contained 50 mM Tris (pH 7.4), 150 mM NaCl, 1% Triton X-100, 1% sodium deoxycholate, 0.1% SDS, sodium orthovanadate, sodium fluoride, EDTA and leupeptin. Tissue was lysed by homogenization for 30 min followed by centrifugation at 14,000 rpm for 30 min. The supernatant was collected and protein concentration quantified using Bicinchoninic Acid Assay (BCA) (Pierce Chemical Co., Rockford, USA) prior to Western Blotting. Protein samples (20 μg) were denatured for 4 min at 95°C in sample buffer. Electrophoresis was performed in 10% SDS-PAGE, followed by protein transfer to nitrocellulose membrane (Whatman Co, UK). The membrane was blocked in 5% non-fat dry milk in TBST (10 mM Tris-HCl, pH 7.5, 150 mM NaCl, 0.05% Tween-20) overnight at 4°C followed by incubation in primary rabbit-anti-rat HMGB1 polyclonal antibody (dilution 1:1000, Abcam, NV, USA). Using anti-rabbit IgG-HRP secondary antibody (dilution 1:15000, Jingmei, Shanghai, China) for 1 hour at room temperature, the probed protein was detected with the ECL chemiluminescence system (Bestbio, Shanghai, China). Blots were quantified using Image- Pro-Plus® Software.

#### ELISA for TNFα and IL-6 levels in serum

TNFα and IL-6 levels in serum were determined using a ELISA kit (R&D system Inc, MN, USA) and expressed in pg/ml.

### Histopathology examination and Immunohistochemistry

Small portions (0.5 cm × 0.5 cm) of liver samples were fixed immediately in 10% buffered paraformaldehyde (pH 7.2) and embedded in paraffin. These portions were cut into 4 μm thick sections and stained using hematoxylin and eosin (H & E). For the severity of hepatic injury, several areas of hematoxylin and eosin-stained tissue were examined under high-powered field microscopy for signs of liver injury including condensation of nuclei (nuclear pyknosis), loss of hepatocellular borders, areas of necrosis, and neutrophil infiltration. Further samples were examined after removal of paraffin, re-hydration and submersion in an antigen retrieval buffer (10 mM sodium citrate; pH 6.0) using a microwave oven at 95–100°C for 5 min. This was followed by incubation at room temperature with 3% hydrogen peroxide to deactivate endogenous peroxidases. Nonspecific reactivity was blocked using 2% BSA at room temperature for 30 minutes. Incubation with Anti-HMGB1 primary rabbit-anti-rat Ab (dilution 1:100; Abcam, NV, USA), Anti-HNE Ab (dilution 1:100; R&D, MN, USA) or Anti 8-OH-G-G Ab (dilution 1:100; Abcam, NV, USA) occurred overnight at 4°C. After washing with PBS, a polymer enhancer and a polymerized anti-rabbit or anti-mouse Secondary Ig G (dilution 1:200, Jingmei, Shanghai, China) labelled with horseradish peroxidase was applied. HMGB1, HNE or 8-OH-G were visualized as buffy granules in the cytoplasm using a DAB kit (Fujian Maixin Biological Technology, Fujian, China).

### Data analysis

Data analysis was performed using the Prism 4.0 statistical software package (Graph-Pad Software, San Diego, CA). Data are expressed as mean ± SEM. Analysis of variance (ANOVA) with Bonferroni’s multiple comparisons test was used for statistical analysis to compare values among all groups. Statistical differences were considered significant if the *p* value was less than 0.05.

## Results

### Intraperitoneal injection of Hydrogen-enriched saline protects against liver I/R injury

**Figure 2:**
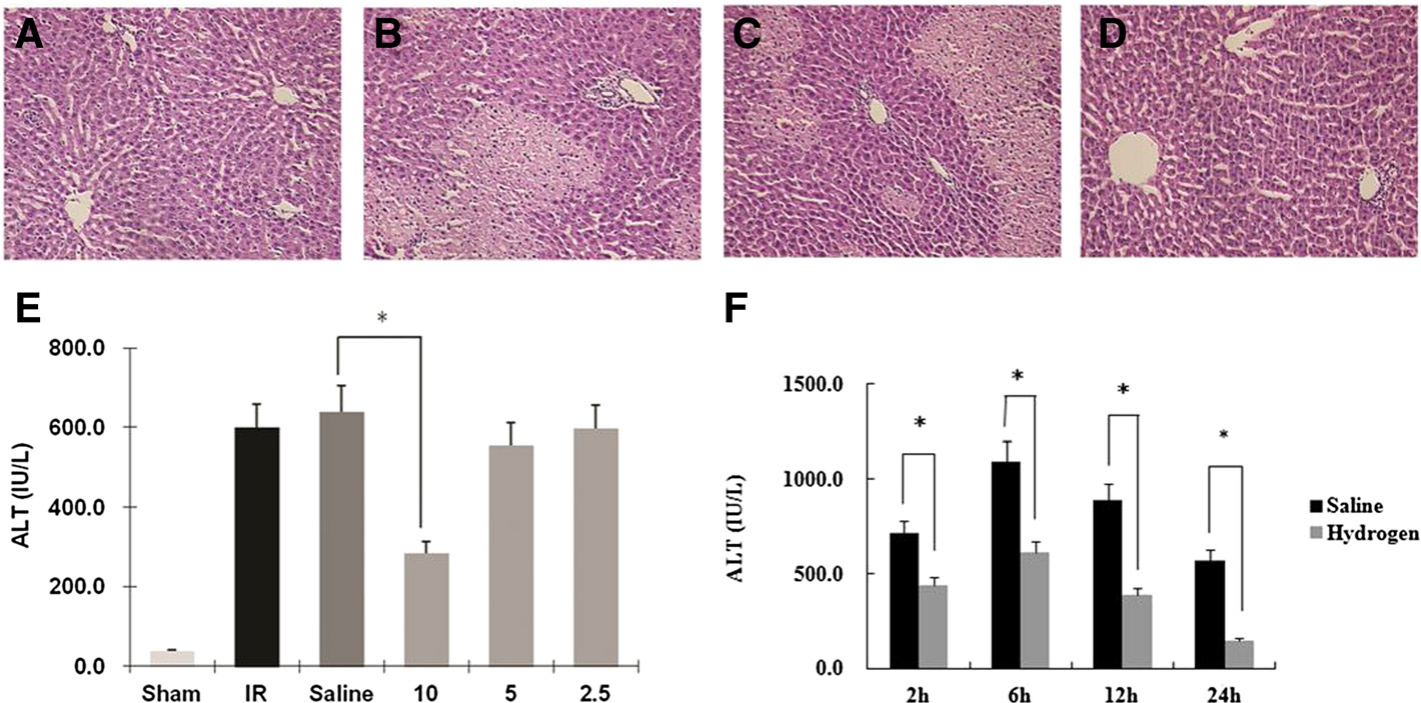
**Hydrogen-enriched saline treatment protects liver against I/R injury.** Male Sprague Dawley rats were subject to partial warm liver ischemia/reperfusion injury with intraperitoneal injection of either normal saline or hydrogen enriched saline at dose of 2.5, 5 or 10 ml/kg 10 minutes before reperfusion. Liver damage was assessed 2 hours after reperfusion with paraffin sections stained with H&E (original magnification × 200) and serum ALT level measurement. An example of microphotograph from **(A)** a sham-operated animal**, (B)** an animal undergoing 60 minutes of ischemia followed by 2 hours of reperfusion without any treatment, **(C)** with normal saline (10 ml/kg), or **(D)** hydrogen-enriched saline (10 ml/kg). **(E)** The dose-response (2.5-10 ml/kg) of hydrogen-enriched saline treatment and 10 ml/kg saline treatment on serum ALT release. Sham-operated animals underwent laparotomy only. Mean ± SEM (n = 8), *p < 0.05. **(F)** The time-course of liver damage assessed with ALT measurement at 2, 6, 12 and 24 hours after reperfusion with normal saline or hydrogen enriched saline treatment (10 ml/kg).

The histology of liver section from the sham group appeared normal (Figure 2A). Positive control animals showed histological evidence of tissue necrosis 2 h after reperfusion (Figure 2B). Severe sinusoidal congestion, neutrophil infiltration and hepatocellular necrosis was readily seen. Histological changes of similar magnitude were found in the saline treated group (Figure 2C). In contrast, histological evidence of tissue damage following hydrogen-enriched saline treatment was greatly reduced and cell necrosis was not easily detected (Figure 2D) at 2 hours post reperfusion.

Changes in serum ALT reflected a similar pattern. 60 minute of ischemia followed by 2 hours of reperfusion significantly increased serum ALT level to 600 ± 62.9 IU/l compared with 38.8 ± 2.5 IU/l found in the sham treated animals. A similar magnitude of ALT increase was seen following normal saline with I/R treatment. 10 ml/kg hydrogen-enriched saline treatment significantly reduced ALT to 284.1 ± 34.0 IU/l after 2 hours of reperfusion (*p* < 0.05). However, 2.5 and 5 ml/kg hydrogen-enriched saline did not significantly reduce ALT rise (Figure 2E).

At each time point, 10 ml/kg Hydrogen-enriched saline treatment significantly reduced ALT rise, as compared to treatment with 10 ml/kg 0.9% saline (Figure 2F).

### Hydrogen-enriched saline protects against I/R-induced peroxidation injury

**Figure 3:**
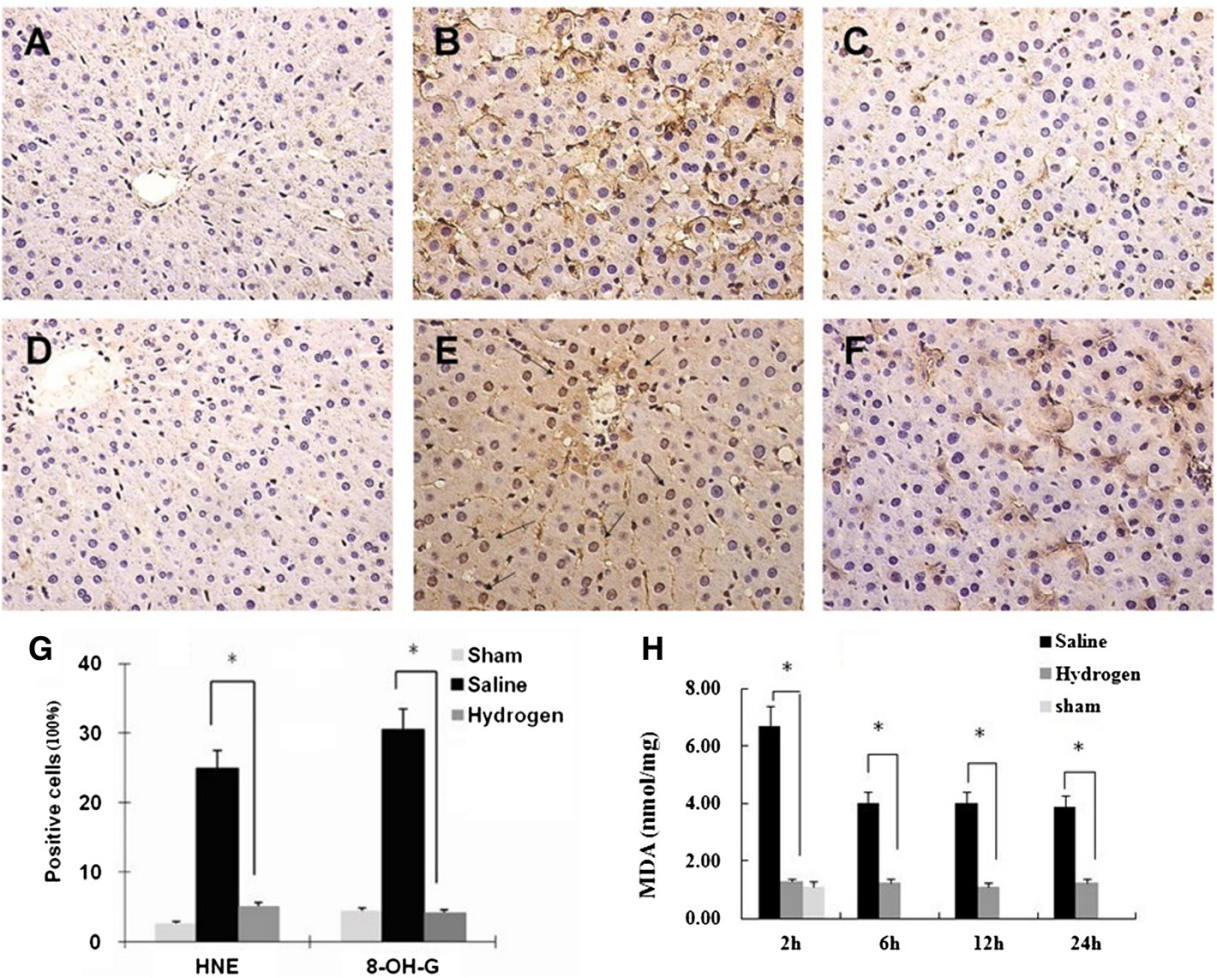
**Hydrogen-enrich saline inhibited hepatic peroxidation induced by ischemia/reperfusion.** Male Sprague Dawley rats were subjected a partial warm liver ischemia/reperfusion injury with intraperitoneal injection of either normal saline or hydrogen enriched saline at dose of 10 ml/kg 10 minutes before reperfusion. Liver damage was assessed 2 hours after reperfusion using immunohistochemistry staining of paraffin sections for HNE or 8-OH-G (original magnification × 400), and hepatic tissue MDA measurement. An example of microphotograph stained for HNE (brown precipitation at cellular membrane) and nucleus (blue) from **(A)** a sham-control, **(B)** 2 after reperfusion with normal saline, or **(C)** hydrogen enriched saline treatment. An example of microphotograph stained for 8-OH-G (brown precipitation at nucleus) and nucleus (blue) from **(D)** a sham-control, **(E)** 2 hours after reperfusion with normal saline, or **(F)** hydrogen enriched saline treatment. **(G)**. Percentage of peroxidative cells was significantly lower in 10 ml/kg hydrogen enriched saline treatment in comparsion of 10 ml/kg saline treatment. (*P < 0.05) **(H)** MDA contents obtained from liver sections subjected to reperfusion for 2, 6, 12, and 24 hours after 60 minutes ischemia with normal saline or hydrogen enriched saline treatment. Sham-operated animals underwent laparotomy only. Mean ± SEM (n = 8), *p < 0.05.

Using Immunohistochemistry, few HNE positive staining cells were seen in the naïve control (Figure 3A) while HNE expression was readily observed in the I/R group (Figure 3B). The immunoreaction was notably reduced in the hydrogen enriched saline treatment group (Figure 3C), with a similar effect seen for 8-OH-G immunoreactivity (Figure 3D-F).

The number of terminal positive cells was counted in six random highpowered (1 × 400) microscopic fields and the oxidative index was defined as the number of positive cells with brown precipitation at cellular membrane or nuclear in every 100 counted cells using Image-Pro-Plus Software.

MDA levels increased significantly in liver tissue obtained from normal saline treated animals. However, hydrogen-enriched saline treatment maintained the MDA content at a significantly lower level at all time points, when compared to those in the I/R group (p < 0.05) (Figure 3H).

#### Hydrogen-enriched saline prevents I/R-induced over-expression and release of HMGB1

Liver I/R increases HMGB1 levels both locally and systemically [7,8]. To determine if hydrogen-enriched saline can modulate this process, immunohistochemistry and western blotting for HMGB1 was performed on both liver sections and on serum obtained from animals in each treatment group.

**Figure 4:**
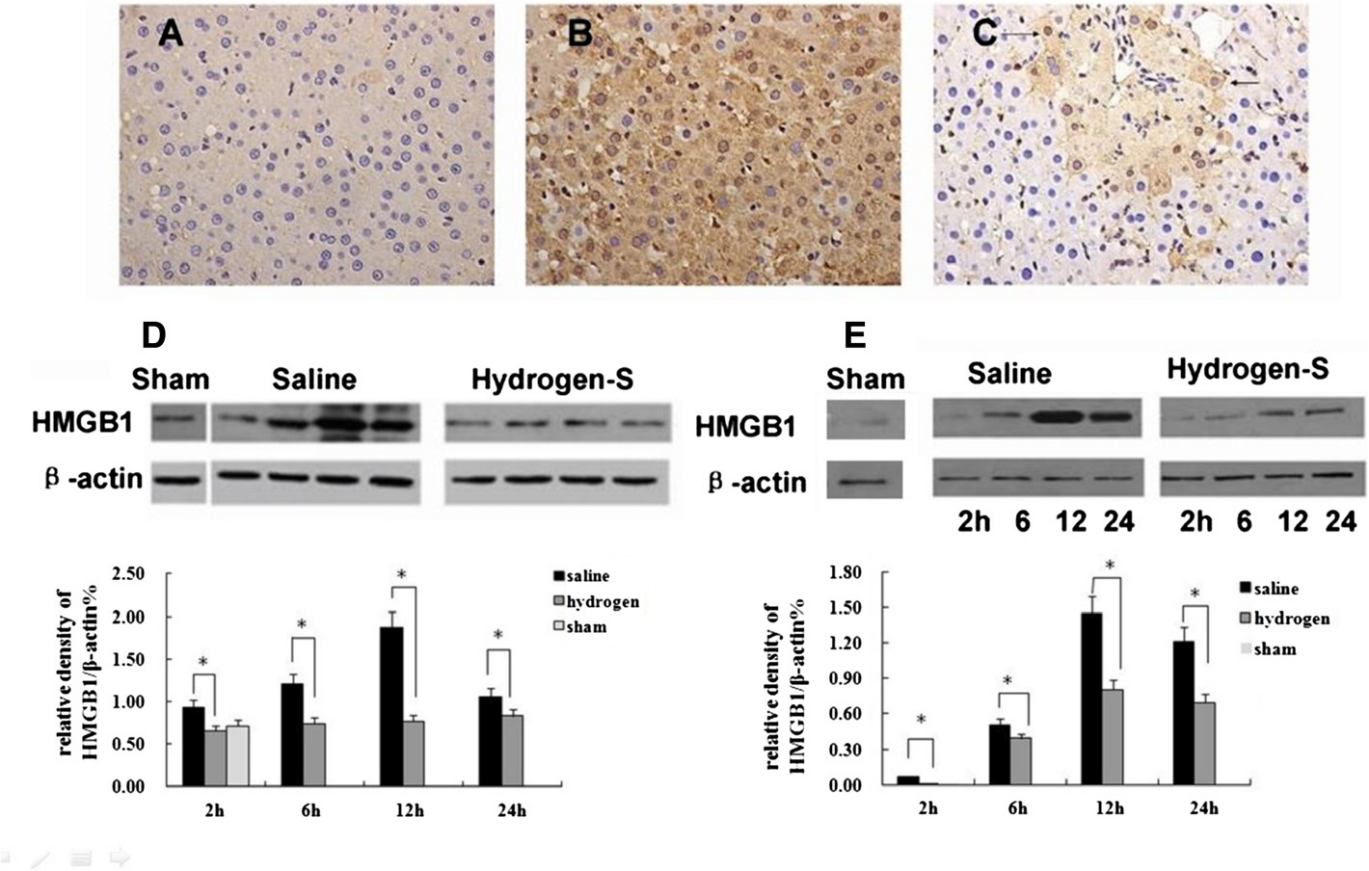
**Hydrogen-enriched saline prevents liver ischemia/reperfusion-induced HMGB1 over-expression and release.** Male Sprague Dawley rats were subject to partial warm liver ischemia/reperfusion injury with either intraperitoneal injection of normal saline or hydrogen enriched saline at a dose of 10 ml/kg, 10 minutes before reperfusion. HMGB1 over expression in the liver were assessed by immunostaining and western blot. Serum levels were assayed by western blot. Examples of microphotographs stained for HMGB1 (seen as a brown precipitation both within the cytoplasm and nucleus, original magnification × 400): **(A)** sham-control, **(B)** 2 hours after reperfusion with normal saline, or **(C)** 2 after reperfusion with hydrogen enriched saline.** (D)** Western blot analysis for HMGB1 was performed on both liver sections and **(E)** serum samples collected from rats 2, 6, 12 and 24 hours after reperfusion. Sham-operated animals underwent laparotomy only. Mean ± SEM (n = 6); *p < 0.05.

Immunohistochemistry showed that in sham control treated rats, no significant HMGB1 was detected in hepatocyte nucleus or cytoplasm (Figure 4A). The I/R group showed increased levels of HMGB1 located within both hepatocyte nucleus and cytoplasm (Figure 4B). The hydrogen-enriched saline treatment group showed reduced the I/R-induced HMGB1 over-expression and shuttling (Figure 4C). These changes in HMGB1 expression were confirmed by western blot (Figure 4D). Additionally, serum levels of HMGB1 were increased in the saline treated group, but were significantly lower in the hydrogen-enriched saline treated group (Figure 4E).

#### Hydrogen-enriched saline decreases liver I/R-induced inflammatorycytokine production

**Figure 5:**
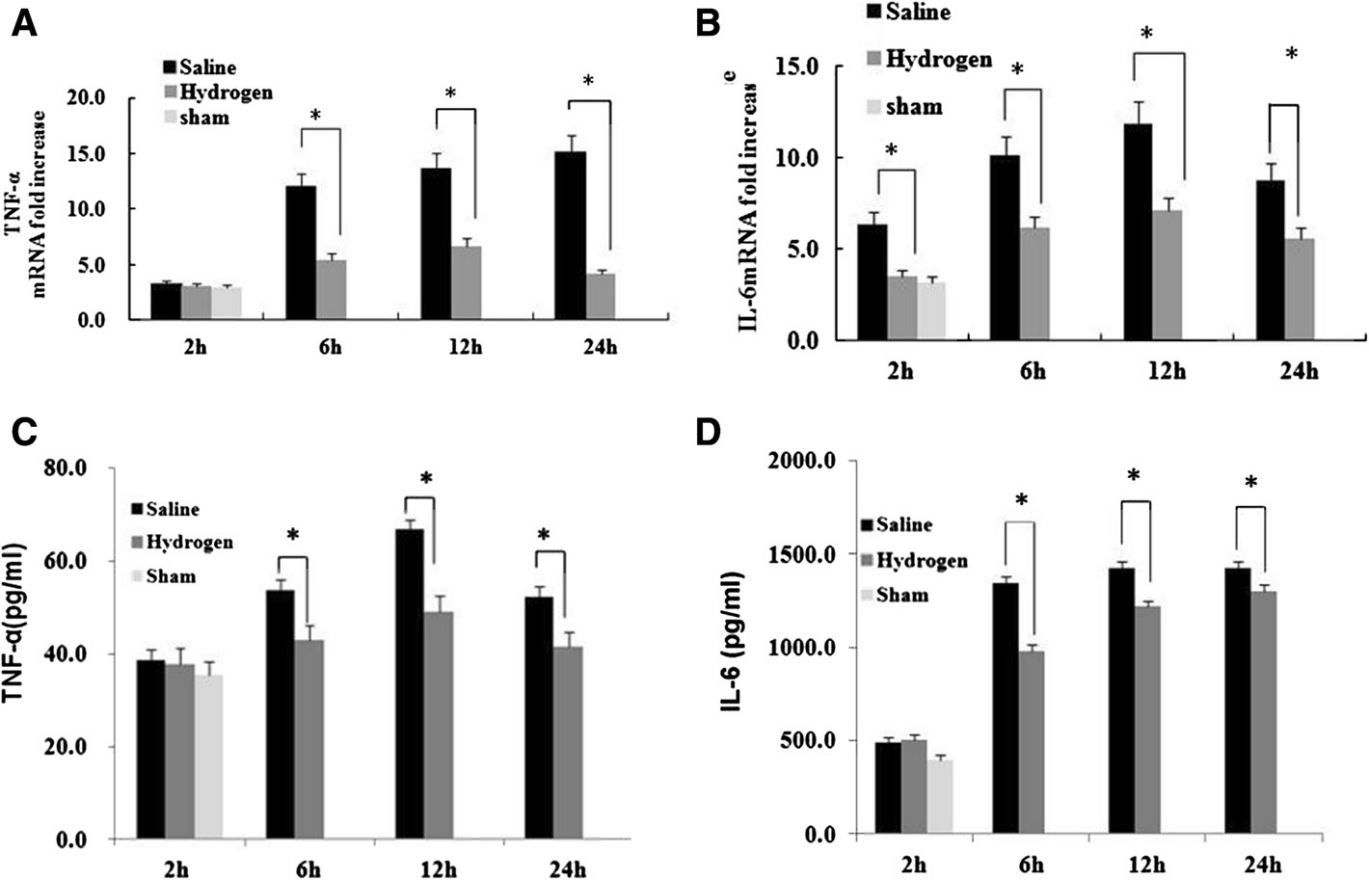
**Hydrogen-enriched saline attenuates TNFα and IL-6 mRNA expression in the liver and reduces serum TNFα and IL-6 levels in serum following liver ischemia reperfusion injury.** Male Sprague Dawley rats were subject to partial warm liver ischemia/reperfusion injury with intraperitoneal injection of either normal saline or hydrogen enriched saline at dose of 10 ml/kg 10 minutes before reperfusion. **(A)** TNF-α and **(B)** IL-6 mRNA expression in the liver were measured by quantitative RT-PCR analysis at 2, 6, 12 and 24 hours after reperfusion and compared to the baseline levels prior to IRI. **(C)** Serum TNF-α and **(D)** IL-6 proteins levels were assessed by enzyme-linked immunosorbent assay. Mean ± SEM (n = 6 -8); *p < 0.05.

Inflammatory cytokines play a critical role in the pathophysiology of liver I/R injury. We used RT-PCR to measure the level of TNF-α and IL-6 gene transcription in the liver with ELISA to measure gene translation products in the serum. TNF-α and IL-6 mRNA was expessed significantly less in the in the livers of hydrogen-enriched saline treated animals, as compared to those treated with normal saline at 2, 6, 12 and 24 hours after reperfusion (Figure 5A and B). Similarly, serum levels of TNF-α and IL-6 were significantly lower in the hydrogen-enriched saline treated animals as compared to the normal saline group (Figure 5C, D).

## Discussion

In this study we have shown that the administration of hydrogen-enriched saline prior to the reperfusion stage in a surgical model of partial ischemia-reperfusion injury attenuates hepatic damage and dysfunction. This is associated with reduced HMGB1 and pro-inflammatory cytokine production. We hypothesise that this is secondary to reduced ROS generation and oxidative stress in the hydrogen-enriched saline treatment group.

Although reperfusion after sustained ischemia salvage tissue, the reperfusion itself paradoxically induces injury (“reperfusion injury”). It is now well recognized that a protective stimulus can be applied at the onset of reperfusion, thereby attenuating reperfusion injury. This is known as postconditioning. It has been investigated most extensively in the heart but has also been described in the liver [22]. The aetiology is complex and multifactorial involving tissue damage secondary to ATP depletion during hypoxia, followed by further cell injury occurring after the resolution of hypoxia and return of perfusion [23]. Although controversial, both stages are considered to independently mediate tissue damage via the production of directly injurious reactive oxygen species (ROS) [24], as well as substances that modulate a local and systemic inflammatory response.

Oxidative stress can be defined as a disturbance in the balance between the production of ROS (with strong cellular oxidizing potential) and antioxidant defences [25]. It plays an important role in the pathogenesis of various hepatic disorders [26], including I/R injury. ROS generated intracellularly include the superoxide anion radical (O2^**.-**^), hydrogen peroxide (H_2_O_2_), hypochlorous acid (HClO), hydroxyl radical (OH^**.**^), and singlet oxygen (^1^O_2_). These agents are produced as a consequence of normal mitochondrial processes [27], and some behave as endogenous intracellular signaling molecules at low concentrations. However, the strongest of the oxidant species, the hydroxyl radical (^**.**^OH), is highly toxic and is not formed by any enzymatic process, but rather from H_2_O_2_ in the presence of divalent metal ions via the Fenton reaction. It reacts almost instantaneously with many cellular components, including the polyunsaturated fatty acids of membrane lipids, nucleic acids, and proteins. No known detoxification system exists and scavenging (^**.**^OH) is critical to prevent nuclear DNA and protein disorganization as well as lipid peroxidation [28]. Lipid peroxidation can disrupt cellular membrane integrity leading to changes in its fluidity and permeability [29]. In addition, lipid peroxides degrade to reactive aldehyde products, including malondialdehyde (MDA) and 4-hydroxyl-2-nonenal (HNE) [30,31].

The hydrogen molecule has antioxidant properties. It has been demonstrated previously that liver IRI can trigger a cascade of innate-dominated pro-inflammatory immune responses that activate an adaptive immune response, culminating in systemic inflammation [3,32]. TLR4 activation by endogenous and exogenous ligands has been confirmed to stimulate the production of pro-inflammatory cytokines including TNF-α and IL-6. These mediate cell death and can further enhance the pro-inflammatory response [33,34]. Indeed, recent studies have shown that endogenous TLR4 ligands, including HMGB1 generated during liver IRI, can trigger a local inflammatory reaction that culminates in hepatocellular damage. Similarly, blocking HMGB1 production and release can effectively minimize liver damage from ischemia [7].

However, recent studies using hydrogen treatment in liver IRI have mainly focused on its anti-oxidative rather than anti-inflammatory action, with little published work having explored HMGB1's role in this process [10]. We have shown in the current study that hepatic IR injury triggers the release of HMGB1 in liver tissue and its subsequent release into serum, and that intraperitoneal hydrogen-rich saline can modulate this.

There is increasing evidence that extracellular HMGB1 acts as an inflammatory mediator in ischemia, hemorrhagic shock, noninfectious hepatitis, and peripheral tissue trauma [35,36]. HMGB1 is actively secreted by activated macrophages [37] and passively released through the porous membrane of cells undergoing necrosis. It has been shown to mediate lethality in sepsis models [38,39]. Recent studies show that HMGB1 is mobilized and released in response to hypoxia, suggesting that the actions in IR occur before cell death [8]. HMGB1 release from cultured hepatocytes is an active process regulated by ROS including H_2_O_2._ Furthermore, HMGB1 release from hepatic cells can occur without causing measurable cell death, and HMGB1 release is mediated by NADPH oxidase or TLR4 signal transduction in a ROS-dependent manner [8]. However, the exact mechanism of ROS regulating HMGB1 release remains unknown. In our study, the results of immunohistochemistry showed that HMGB1 was not detected in the sham group, while it is found both in nucleus and cytoplasm of hepatocytes induced by ischemia reperfusion. Meanwhile, much less HMGB1 was detected in hydrogen-enriched saline treated animals compared with saline group. The results indicated that the ^**.**^OH attacked the DNA and lead to exposure antigen recognition site of HMGB1. The freed HMGB1 moved from nucleus to cytoplasm through increased permeability nuclear membrane induced by peroxidation. Finally, they were released to extracellular and acted as an initiator of inflammation. Therefore, our results suggest that, during hepatic I/R, systemic HMGB1 levels are associated with the degree of hepatocellular peroxidation, indicating that HMGB1 is a marker of cell damage that reflects the integrity of cellular structure.

Hydrogen has a powerful ability to penetrate biomembranes and diffuse into the cytosol, mitochondria and nucleus thereby effectively reducing the hydroxyl radical, the most cytotoxic of reactive oxygen species. Its ability to protect nuclear DNA and cell membrane suggests that it can reduce oxidative stress induced cellular injury and the subsequent inflammatory response [14]. Hydrogen gas cannot be produced by the human body since mammalian cells lack the hydrogenase activity. However, it is continuously produced by colonic bacteria in the body and normally circulates in the blood, so it is physiologically safe for humans to inhale hydrogen at a relatively low concentration. Medical use of hydrogen in the past was limited to test the effects of antibiotic therapy. Previously, other therapeutic strategies for scavenging reactive oxygen species seemed promising in animal models but most of them failed in human clinical trials [18]. This study demonstrates that hydrogen-rich saline protects the liver against cellular injury and organ dysfunction through a mechanism that reduces the impact of oxidative stress and associated inflammation. Its ease of preparation and administration, and its favourable safety profile make hydrogen-enriched saline an attractive and potentially clinically useful tool.

Certain limitations to our study should be considered. The intraperitoneal administration of our treatment agent is not a clinically used modality, although intravenous administration has been safely employed by other researchers in animal models of organ I-R (9). In addition, blood and tissue levels of hydrogen were not determined so we do not know the bioavailability of this route of administration.

## Conclusions

In summary, this study documents the ability of hydrogen-enriched saline to protect against hepatic I/R in an animal model. This protection was associated with a reduction in both oxidative stress and inflammatory injury and is in contrast to the lack of protective effect seen after saline treatment. We have shown reduced peroxidation injury secondary to oxidative stress, and attenuation of the release of inflammatory mediators. We believe that this is an important association which merits further investigation of the possible underlying mechanisms since hydrogen-enriched saline may have potential as a novel antioxidant and anti-inflammatory agent in the clinical setting.

## Competing interests

The authors declare that they have no competing interests.

## Authors’ contributions

YL: animal preparation, performance of experimental work, preliminary data analysis, and drafting of the manuscript. LY: analysis of mechanical and histological data, statistical analysis, and writing of the manuscript. KT: performance of experimental work, analysis of mechanical and histological data and statistical analysis. MV and DL: performance of experimental work and analysis of data. XS: preliminary data analysis, and drafting of the manuscript. Sun contributed to the experimental design. MI :experimental design and writing of the manuscript. DM: the experimental design and writing of the manuscript. WY: experimental design, supervision of experimental work, statistical analysis, writing of the manuscript, and supervision and overview of entire project. All authors read and approved the final manuscript.

## Pre-publication history

The pre-publication history for this paper can be accessed here:

http://www.biomedcentral.com/1471-230X/14/12/prepub
